# Adipocytes and Macrophages Interplay in the Orchestration of Tumor Microenvironment: New Implications in Cancer Progression

**DOI:** 10.3389/fimmu.2017.01129

**Published:** 2017-09-19

**Authors:** Luís Henrique Corrêa, Rafael Corrêa, Cecília Menezes Farinasso, Lívia Pimentel de Sant’Ana Dourado, Kelly Grace Magalhães

**Affiliations:** ^1^Laboratory of Immunology and Inflammation, Department of Cell Biology, University of Brasilia, Brasilia, Brazil

**Keywords:** adipocytes, cancer, tumor microenvironment, obesity, inflammation, tumor-associated macrophages, tumor-associated adipocytes, macrophage polarization

## Abstract

Inflammation has been known as one of the main keys to the establishment and progression of cancers. Chronic low-grade inflammation is also a strategic condition that underlies the causes and development of metabolic syndrome and obesity. Moreover, obesity has been largely related to poor prognosis of tumors by modulating tumor microenvironment with secretion of several inflammatory mediators by tumor-associated adipocytes (TAAs), which can modulate and recruit tumor-associated macrophages. Thus, the understanding of cellular and molecular mechanisms that underlay and link inflammation, obesity, and cancer is crucial to identify potential targets that interfere with this important route. Knowledge about the exact role of each component of the tumor microenvironment is not yet fully understood, but the new insights in literature highlight the essential role of adipocytes and macrophages interplay as key factor to determine the fate of cancer progression. In this review article, we focus on the functions of adipocytes and macrophages orchestrating cellular and molecular mechanisms that lead to inflammatory modulation in tumor microenvironment, which will be crucial to cancer establishment. We also emphasized the mechanisms by which the tumor promotes itself by recruiting and polarizing macrophages, discussing the role of adipocytes in this process. In addition, we discuss here the newest possible anticancer therapeutic treatments aiming to retard the development of the tumor based on what is known about cancer, adipocyte, and macrophage polarization.

## Introduction

### The Tumor Microenvironment

Tumor is not composed only of the cancer cells. Instead, there is a dynamic and mutualist relationship between tumor cells and the surrounding stroma (1). Studies have shown an intimate relationship between tumor mass cells and their extracellular matrix correlating this interaction with cancer establishment, progression, and metastasis. The extracellular matrix is responsible not only for the structural support of the cells but also for storing important signaling molecules, such as chemokines (2).

Cancer cells recruit non-malignant cells into their microenvironment, where reciprocal modulation takes place: tumor cells configure several cell types and change their metabolism to favor tumor promotion, resulting in a more anti-inflammatory environment and the avoidance of antitumor immune cells. Each non-malignant cell type exerts a function in the microenvironment in order to support tumor growth and development, which leads to metastasis (3).

An important component recruited for the tumor stroma is the tumor-associated macrophage (TAM). These cells have an extremely important action for tumor progression (4). Macrophages are recruited and polarized to the M2 configuration in which they act to compose the tumor microenvironment mediating an immunosuppression phenome. TAMs are chemoattracted to the microenvironment mainly by chemokines such as CCL2, which are produced in high quantities by malignant cells (5). In the tumor site of action, TAMs produce cytokines such as IL-10, IL-4, and IL-13 that will act to repair tissues and attract other auxiliary cells that can activate TAM and hence, promoting tumor growth (6).

Similarly, neutrophils are also part of the tumor microenvironment, being an important source of cytokines and reactive oxygen species (ROS). T cells are also found acting in an immunosuppressive manner, secreting TGF-β and IL-10 that inhibit the antitumor activity of cytotoxic T-cells and natural killer cells (2). Fibroblasts associated with the tumor have a fundamental participation in the production of vascular endothelial growth factor (VEGF), that induces angiogenesis, along with vascular endothelial cells that are responsible for forming blood vessels that support tumor growth and metastasis (1).

In addition to immunological cells, the tumor microenvironment is composed of adipose tissue. Adipose tissue has been widely studied and it has a great potential as an endocrine organ (7), since this tissue is known to be able to secrete a huge variety of different adipokines, such as leptin, hepatocyte growth factor, and adiponectin. Adipocytes can stimulate the growth and the survival of tumor cells (8) and then are able to perform a crosstalk with the surrounding cells, releasing fatty acids that can be metabolized by tumor cells and used as energy source.

Tumor-associated adipocytes (TAAs) assist in the recruitment of macrophages to the microenvironment and their polarization to the alternative M2 configuration *via* CCL2, IL-1β, and CXCL12 (9). Recently, Nishimoto et al. showed that macrophage accumulation in the microenvironment is due to not only the secretion of CCL2 by adipocytes but also the release of degenerate adipocyte DNA (10).

The complex cellular arrangement that constitutes the tumor microenvironment is dynamic. Malignant cells are dependent on the cells of the immune system and fat cells to maintain and develop. Therefore, including all factors, the tumor microenvironment possesses the proliferative abilities to evade suppression, resist cell death, develop replicative immortality, induce angiogenesis, and stimulate not only invasion but also metastasis. A variety of cells and cytokines can be found in tumor microenvironment and they have different implications in cancer progression (Table 1).

**Table 1:** Cellular composition of the tumor microenvironment and the implication of the cytokines secretion in the tumor development.

Cell type	Cytokines	Function
Classical macrophages (M1)	IL-1β, IL-12, TNF-α	Proinflammatory M1 profile. It makes the environment prone to inflammation and tumor cells elimination can be triggered by secretion of IL-1β, IL-12, and TNF-α cytokines
Alternative macrophages (M2)	IL-4, IL-10, TGF-β	Anti-inflammatory M2 profile. It maintains the tumor by cytokines secretion involved in tissue regeneration and remodeling, such as TGF-β, IL-10, IL-4, increasing the tumor cells viability, facilitating tissue invasion by tumor cells, and metastasis
Adipose cells	IL-6, IL-10, IL-13, IL-33, TGF-β, TNF-α	Adipose tissue can favor tumor growth and development by producing anti- and proinflammatory mediators in a deregulated manner. Proinflammatory cytokines, such as IL-6 and TNF-α, are secreted, promoting cell proliferation and angiogenesis. Other cytokines, such as IL-13, IL-10, and TGF-β are also produced and their role is to maintain the tumor, regenerating tissues, and inhibit anticancer immune cells recruitment
CD8+ T cells	IFN-γ	CD8+ T secrete large amounts of cytotoxic granules and IFN-γ, which polarizes macrophages into the proinflammatory M1 profile and inhibits Treg lymphocytes
CD4+ T and Treg cells	IL-10, TGF-β	These cells can assist in the suppression of other immune cells: IL-10 and TGF-β secretion inhibits antitumor NK and CD8+ T cells
Neutrophils	TGF-β, TNF-α, IL-4, IL-6, IL-1β	Neutrophils have both pro- and antitumor functions. They help to maintain the M2 profile by producing IL-4 and TGF-β, which support the tumor cells growth. These cells can also play an anticancer role, secreting TNF-α, IL-6 and IL-1β to prolong the CD8+ T population

### Tumor-Associated Adipocytes

Cancer cells usually activate wound-healing response of the host to generate a supportive microenvironment to favor tumor development (4). It has been described that cancer progression is promoted by stromal cells, such as cancer-associated fibroblasts (CAFs) or TAMs, which secrete extracellular matrix components, chemokines, and growth factors that stimulates cell migration and metastasis (2). The contribution of fibroblasts, endothelial, and inflammatory cells to cancer establishment has been extensively studied (1). However, the comprehension of the real role of adipocytes in cancer environment is still poorly understood since adipocytes were mainly considered an energy storage depot until recently. Only recently, adipocytes started to be beheld as endocrine and inflammatory cells producing hormones, growth factors, cytokines, and other molecules such as adipokines (5). Moreover, early local tumor invasion results in immediate proximity of cancer cells to adipocytes in breast cancers (3). Hence, adipocytes represent extraordinary candidates to regulate tumor behavior through heterotypic signaling processes.

The interaction of adipose tissue with tumor cells is widely described and characterized as poor prognosis. Adipose tissue is a very dynamic and complex tissue, mostly composed of adipocytes but it also contains macrophages, lymphocytes, endothelial cells, pericytes, and adipocyte progenitor cells. Cancer development and progression is improved by both adipocytes and stromal vascular cells in adipose tissue (11). The enhance of the adipose tissue, leading to a series of metabolic deregulations, is a key characteristic of obesity and a greater risk of cancer (12). It is known that inflammation is closely linked to obesity. Likewise, obesity is intimately related to cancer evolution. Thus, obesity, inflammation, and tumor comprise an important triangle that can orchestrate a critical modulation in tumor microenvironment through the action of its mainly players: adipocytes and macrophages.

Adipocytes produce inflammatory cytokines, such as TNF-α, IL-6, IL-1β, and CCL2 (9, 13), which leads to directly inflammatory cell (e.g., lymphocytes and macrophages) recruitment, infiltration and accumulation in adipose tissue, and establishes a state of low grade chronic inflammation (14, 15). Macrophage infiltration was reduced in CCL2 knockout mice, or its receptor CCR2, as well as the obesity-induced inflammation in visceral adipose tissue (16, 17). Visceral adipose tissue from obese people are frequently associated with increased levels of CCL2, TNF-α, IL-1, IL-6, and inducible nitric oxide synthase (iNOS) (18). In subcutaneous fat pads, both macrophage recruitment and expression of CCL2 were elevated when mice was ovariectomized, suggesting that increase inflammation in postmenopausal breast tissue may be due to CCL2 (18). Moreover, the amount of CCL2 produced is increased due to recognition of cell-free DNA (cfDNA) from degenerate adipocytes. Obese mice showed an increase in cfDNA release leading to a higher accumulation of macrophages, which aggravates inflammation (10). In response to CCL2 and IL-1β, recruited macrophages secrete CXCL12 in order to induce angiogenesis and support the expanding tissue (9).

Reactive oxygen species are also involved in the tumorigenic process. When a state of chronic inflammation is established in adipose tissue, the generation of ROS is observed (19). In low concentrations, ROS has mitogenic properties and can be considered as a tumor promoter (20).

Comparing adipose tissue macrophages (ATMs) and monocyte-derived macrophages (MDMs) from the same obese patients, ATM secreted soluble factors that induce inflammation and lipid accumulation in cancer cells (T47D and HT-29), but MDM did not. Additionally, functional clusters, such as cytokine–cytokine receptor interaction (particularly CXC chemokine) signaling, and cancer-related pathways are overexpressed only in ATM. Gene expression profiles of TAMs were more similar to ATM than MDM. Interestingly, MDM also acquired an ATM phenotype and modulated the secretion of factors by preadipocytes but not by mature adipocytes (21).

White adipose tissue from obese individuals present abundant crown-like structures (CLSs) which are constituted by infiltrating macrophages surrounding dead adipocytes. Enhanced rates of CLS in white adipose tissue are often associated with increased production of the inflammatory mediators in obese individuals. Macrophages from CLS trigger adipocyte cell death, aggravating the tissue inflammatory condition and attracting more infiltrated macrophages to the tumor microenvironment (22). Additionally, obese adipose cells produce the nuclear protein high mobility group box 1 in large quantities (23), a danger signal that attracts and activates immune cells (24).

The initial factors that trigger chronic inflammation of adipose tissue in obesity are unclear. Evidence suggests it might be mediated by hypoxia, necrosis, increased secretion of immunomodulatory factors (such as cytokines, chemokines, hormones, and growth factor), lipolysis, and free-fatty acids release (25).

Tumor-associated adipocytes can be delipidated, shedding elements of adipose tissue into tumor microenvironment. Those adipocyte-derived elements can be used by cancer cells to promote tumor growth. TAA can potentially dedifferentiate into fibroblast-like cells. Free-fatty acids released by adipocytes can be used by cancer cells to fuel its growth and proliferation. In addition, free-fatty acids can activate and modulate other cells such as myocytes, macrophages, and vascular endothelial cells favoring the formation of protumorigenic microenvironment (4, 26). Likewise, CAFs, in conjunction with others stromal cells, promote tumor progression (27). Obese adipose tissue can regulate tumor development in different ways, either by providing energy *via* free-fatty acids or through adipokines, cytokines, or miRNAS. An increasing number of miRNAs has been associated with several diseases, such as cancer, diabetes, and obesity. Recently, Thomou et al. (28) which shows the ability of adipose tissue to regulate gene expression in other tissues through circulating exosomes containing miRNAs showing the enormous influence that adipose tissue can exerts on other tissues by directly silencing genes.

Malignant cells around adipose tissue secrete sufficient amounts of cytokines that will polarize macrophages to the M2 alternative profile, promoting the establishment of an environment more conducive to the development of a tumor (6). Macrophages tend to switch from an initial M1 profile to a later M2 profile (29). In obese mammary adipose tissue, macrophages showed a decreased IL-10 expression and increased iNOS and CD11c expression consistent with a M1 polarization phenotype, on the other hand, the cells showed an increase of CD206 expression, suggesting a mixed polarization phenotype in obese mammary glands (9). The coculture of adipocytes with macrophages (THP-1-derived cells) induces a shift of THP-1 macrophages to a M2 phenotype (30). Since in this experiment there was no contact between the cell types, the findings suggest that the crosstalk is mediated by soluble factors secreted by adipocytes promoting macrophage polarization.

### Tumor-Associated Macrophages

The macrophages polarization occurs through different ligands that act modulating their metabolism. This macrophage plasticity is essential for the establishment of an antitumor immune system functionality. These cells can vary from a configuration that inhibit tumor growth and induce cell death (the classical M1 profile) to a configuration that stimulates cancer progression and tissue repair (the alternative M2 profile) (31).

Cancer development and progression are deeply regulated by immune system. It is known that the immune cells infiltration at the tumor site may affect the progression of malignancy and metastasis (32), where macrophages are the most abundant immune cells in the tumor microenvironment (33). Macrophages are characterized by their plasticity, flexibility and ability to integrate distinct signals from the microenvironment acquiring distinct phenotypes (34). In this context, macrophages can be divided into two subtypes: M1 macrophages, characterized as promoting Th1 response and strong microbicidal and tumoricidal activity; and M2 macrophages that promote Th2 response, tissue remodeling, immune tolerance, and tumor progression (35, 36).

Activated macrophage population phenotype can change occasionally. In obesity, for instance, there is a macrophage phenotype switch from M2 to M1 (37). Contrarily, tumor progression is often associated with macrophage phenotype changes from classically activated (M1) to alternatively activated (M2) (6). Macrophage plasticity occur in consequence of a dedifferentiation of the original cells back to their resting state or the migration of a new population of macrophages into the tissue site to replace the original cells. Other studies have indicated that changes in cell stimuli directly impact the macrophage polarization (M1 or M2) (38). Then, macrophages are capable of repolarizing in response to modifications in the local microenvironment, permitting them to shape the local inflammatory milieu to adjust to different sources of stimuli.

M1 macrophages are stimulated by microbial products or proinflammatory cytokines (IFN-γ and TNF-α) (34). M1 macrophages are known sources of proinflammatory cytokines such as TNF-α, IL-1, IL-6, IL-12, and type I IFN; chemokines such as CXCL1–3, CXCL-5, and CXCL8–10; and high antigen presentation function, nitric oxide, and ROS generation (39). Classically activated macrophages are deeply involved in the recognition and destruction of cancer cells. After recognition by M1 macrophages, tumor cells can be killed through several mechanisms, which include contact-dependent phagocytosis and cytotoxicity (40).

M2 macrophages are anti-inflammatory cells which aid in the process of angiogenesis and tissue repair. These cells are characterized by the upregulation of Dectin-1, mannose receptor, scavenger receptors, CD163, CCR2, CXCR1, and CXCR2, in addition to the production of large quantities of IL-10 and other anti-inflammatory cytokines (35, 41). The expression of IL-10 by M2 macrophages promotes a Th2 response, and Th2 cells, in turn, upregulate the production of IL-3 and IL-4. Furthermore, M2 macrophages are not capable of efficient antigen presentation (42, 43). M2 macrophages have different subsets, each induced by a different set of molecules and different activation responses. For this, M2 macrophages can be further divided into subgroups called M2a, M2b, M2c, and M2d. M2a macrophages are induced by the Th2cytokines, IL4 and IL13; their function is to activate Th2 response by producing IL-10, TGF-β, and IL-1ra, with the purpose of promoting type II inflammation to kill parasites. M2b cells are induced by immune complexes, lipopolysaccharides, IL-1R and TLRs ligands; they produce cytokines such as IL-1, IL-6, TNF-α, and IL-10, acting in the metastasis control, suppressing tumor growth and inducing a Th1 response. IL-10, TGF-β, and glucocorticoids can induce macrophages to differentiate to M2c phenotype, which is involved in immune suppression, tissue repair, and matrix remodeling (34, 44). Recent research has demonstrated that TAMs exhibit functions like those of M2 macrophages and can be classified as M2d subtype. M2d or TAMs exhibit functions that may help tumor progression by allowing the growth of new blood vessels growth, which feeds the malignant mass of cells, further promoting their growth (45).

The most important characteristic of TAM is the ability to promote the establishment and progression of cancer. This promotion occurs through secretion of cytokines that can act directly on tumor cells or indirectly, by recruiting auxiliary cells. The alternative M2 configuration is the most abundant cell type present in the tumor microenvironment being directly related to inhibition of proinflammatory cell recruitment and inhibition of polarization to the classic M1 type (46).

Tumor cells produce cytokines responsible for the recruitment and polarization of macrophages to M2 profile such as IL-4, IL-10, IL-13, and TGF-β, as well as chemokines such as CCL2 (MCP-1), CCL3, and CCL14. When infiltrated, macrophages increase the expression of mannose and galactose receptors as well as the production of VEGF, cyclooxygenase-2 (COX-2)-derived prostaglandin E_2_ (PGE_2_), and IL-10, thus rendering the microenvironment against inflammation and favorable to tumor development (47). Moreover, TAM phenotype expresses Arg1 and TGF-β in order to recruit Th2 lymphocytes to the tumor site leading to an increase in IL-4 production and maintenance of the M2 population (7).

Tumor-associated macrophage cells have an immunosuppressive role inhibiting the antitumor response of T cells. At the site of the tumor, the high production of IL-10 derived from TAMs indirectly inhibits T cell activation by down regulating IL-12 production by dendritic cells, which, in turn, prevents the TCD8+ response. Furthermore, IL-10 and TGF-β production by TAM promotes induction of regulatory T cells that are installed locally and make the environment even more immunosuppressed (48).

Studies have shown that TAMs do more than rendering an anti-inflammatory microenvironment. TAMs are also related to cancer promotion, angiogenesis induction, as well as tumor cell migration and metastasis (45). Angiogenesis is the first step involved in the occurrence of metastasis. In addition to nourishing tumor mass, TAMs are present throughout the process. TAMs can reorganize the extracellular matrix. Among the list of proangiogenic growth factors secreted by TAMs, the following factors can be highlighted: epidermal growth factor, VEGF, platelet-derived growth factor, migration inhibitory factor, TNF-α, TGF-β, IL-8, and IL-1β, thymidine phosphorylase, and the chemokines CCL2 and CXCL8 (49). These factors can induce the formation of a network in which tumor cells can benefit by receiving nutrients and migrating to other sites.

**Figure 1:**
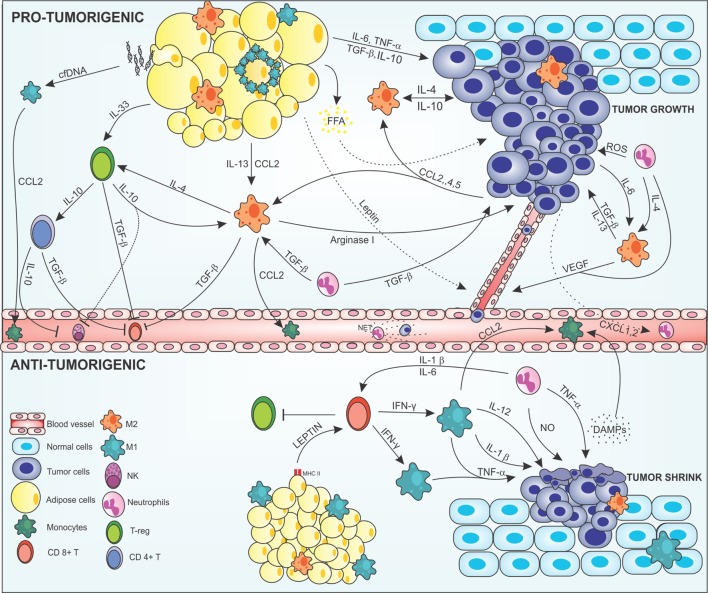
Cross-talk between adipocytes, macrophages, and cancer cells modulating the tumor microenvironment. In the *protumorigenic* state, macrophages are attracted to the tumor site *via* chemokines, such as CCL-2, -4, and -5 produced by malignant cells, resident macrophages, and inflamed adipose tissue. Obese adipose tissue can favor tumor growth and development by producing anti- and proinflammatory mediators in a deregulated way. Proinflammatory cytokines, such as IL-6 and TNF-α, are secreted by adipose cells acting on the tumor modulating the Janus kinase/STAT pathway promoting cell proliferation and angiogenesis. Anti-inflammatory cytokines, such as IL-13, IL-10, and TGF-β are also produced by this tissue and maintain the tumor, regenerating tissues, and inhibiting the recruitment of immune anticancer cells. In addition, inflamed adipocytes produce leptin in large amounts, which act on epithelial cells leading to neovascularization, thus increasing tumor invasion. Degenerate adipose cells attract M1 macrophages that can form a crown-like structure around the inflamed adipose cell to eliminate them. This elimination can lead to FFA release that will nourish the tumor giving energetic support to the malignant cells. In addition, degenerate adipose cells release cfDNA that are recognized by macrophage TLR9 receptors, thus increasing the production of CCL2 in these cells, leading to increased recruitment of monocytes. Macrophages are attracted to the tumor microenvironment and polarized to the M2 alternative profile. Tumor cells produce IL-4, IL-10, and IL-6; adipose cells produce IL-13, IL-10, and TGF-β; and neutrophils IL-4 and TGF-β polarizing and maintaining the macrophages in the M2 configuration. More macrophages are attracted into the microenvironment *via* CCL2, and help to maintain the tumor by producing cytokines that act on tissue regeneration, such as TGF-β, IL-10, IL-4, and arginase-1, increasing the viability of the tumor cells, leading to invasion and metastasis. The M2 configuration regulates the recruitment of immune cells, inhibiting the recruitment of CD8+ T and NK cells *via* TGF-β in addition to maintaining Treg cells by IL-4 production along with production of IL-33 produced by adipocytes. Treg cells act by maintaining the M2 configuration producing IL-10 and TGF-β which also activated CD4+ T activation. CD4+ T cells assist in the inhibition of anticancer cells through IL-10 and TGF-β secretion inactivating NK cells and CD8+ T cells. Neutrophils are also found in the tumor microenvironment. Cancer cells produce CXCL2 in large numbers, recruiting neutrophils that help maintain the M2 profile by producing IL-4 and TGF-β, which also help the growth of tumor cells. Tumor-associated neutrophils are responsible for increasing the mutagenic level of cells by the production of reactive oxygen species (ROS), which cause damage to the DNA of cells, in addition to M2 macrophages, to produce VEGF, and increasing angiogenesis. Neutrophils in blood vessels may form structures such as NETs that help malignant cells stabilize in place and enter the metastatic phase. In the *antitumorigenic* state, normal adipose cells secrete leptin which, together with MHCII, have acted in the recruitment of CD8+ T cells. CD8+ T secrete large amounts of IFN-γ polarizing macrophages into the proinflammatory M1 profile and inhibit Treg cells. M1-like cells act on the secretion of cytokines that have the function of making the surroundings more and more inflamed and destroy tumor cells, such as IL-1β, IL-12, and TNF-α, and recruit more macrophages to the site *via* CCL2. Tumor cells, when destroyed, release DAMPs, which recruiting more macrophages and neutrophils into the site, which aid in inflammation secreting nitric oxide (NO) and causing severe damage to cells. Abbreviations: IFN-γ, interferon γ; IL, interleukin; MHCII, class II major histocompatibility complex; TNF-α, tumor necrosis factor α; TGF-β, transforming growth factor β; CCL2, 4 and 5, chemokine (C-C motif) ligand 2, 4, and 5; CXCL2, chemokine (C-X-C motif); VEGF, vascular endothelial growth factor; NK, natural killer; T-reg, regulatory T cell; CD8 and CD4, cluster of differentiation 8 and 4; cfDNA, cell-free DNA; FFA, free-fatty acid.

Monocytes/macrophages recruitment into tumor microenvironment is mainly controlled by cytokines, chemokines and growth factors produced by stromal and malignant cells (45) (Figure 1). Many cancers like breast (50), prostate (51), ovarian (52), and non-small lung cancers (53), use the release of CCL2 for recruitment and polarization of M2 macrophages (54). Others chemokines such as CXCL12, CXCL8, CXCL1, CXCL13, CCL4, CCL5 (55), CCL17, and CCL22 (56), have been identified in neoplastic tissues as tumor cells products. Moreover, growth factors can be released by tumor cells, like VEGF, placental growth factor, and macrophage-colony stimulating factor (M-CSF). These have also been described as promoters of monocyte/macrophage recruitment (57, 58).

Tumor microenvironmental factors, such as leukemia inhibitory factor, IL-6, and M-CSF, were shown to promote TAM generation. IL-6 can activate the signal transducer and the activator of transcription 3 (STAT3), which is a signal transduction molecule that converges to many oncogenic signaling pathways, and controls the M2-polarization of macrophages as well (59). STAT3 is a constitutive activator in tumor cells (60) and is involved in the initiation and progression of epithelial ovarian carcinoma (61). IL-6/STAT3 pathway further regulates M2 macrophage polarization and promotes tumorigenesis (62).

Macrophage polarization in the tumor inflammatory microenvironment can be regulated by COX-2 and its downstream arachidonic acid metabolite product, PGE_2_ (63). IL-6 can mediate COX-2 expression in a variety of tumors, including colorectal cancer (CRC) (64), breast cancer (65), melanoma (66) and prostate cancer (67); additionally, it induces PGE_2_ production in mesenchymal stem cells, lung cancer, and cervical carcinoma (68, 69). Both molecules contribute to carcinogenesis by stimulating cancer cell proliferation, inhibiting apoptosis, increasing invasiveness, and modulating inflammation and immunity through the induced release of Th2 cytokines, such as IL-10, TGF-β, IL-4, and IL-13, by TAMs and the tumor cells (70).

Analysis of the molecular basis of the TAM phenotype has identified the transcriptional factors NF-kB and hypoxia inducible factor 1 (HIF-1) as central regulators of tumor progression and metastasis (71). NF-kB activation is crucial for cancer-related inflammation and is associated with M1 macrophages during the early phase of tumorigenesis. However, at the late phase of tumorigenesis, macrophages are reprogramed to TAMs or M2-like macrophages presenting low NF-kB activation but increased immunosuppressive capacity (72). Hypoxia executes its effect on macrophages through two isoforms of HIF, HIF-1a, and HIF2 (73). It was demonstrated that high lactic acid produced by tumor cells in this hypoxic environment has a critical function in signaling; specifically, it induces the expression of VEGF and produces hypoxia-inducible factor-1α (HIF-1α) and HIF-2α, which regulate the transcription of genes associated with angiogenesis. Moreover, recent studies also showed tumor hypoxia enhances non-small cell lung cancer metastasis by promoting macrophage M2 polarization through the activation of ERK signaling (29, 74).

Numerous miRNAs have been shown to be highly expressed in polarized macrophages, particularly, miRNA-155, miRNA-125, and miRNA-378 for M1 polarization also as miRNA-let-7c, miRNA-9, miRNA-21, miRNA-146, miRNA147, and miRNA-187 for M2 polarization (75, 76). New studies demonstrated a novel miR-155-mediated mechanism for promoting the M1 phenotype of macrophage polarization. Because miR-155-modified TAMs can regain tumor-killing capacity, this study provides a potential therapeutic approach in cancer treatment (77).

Malignant fibrous histiocytoma amplified sequence 1 (MFHAS1) is a predicted oncoprotein that demonstrates tumorigenic activity *in vivo* (78). In recent studies, it was detected a positive association between MFHAS1 expression in TAMs and human CRC, where CRC cells induced M2 polarization of TAMs through MFHAS1 induction and subsequent STAT6 and KLF4 activation to promote CRC progress. Therefore, the role of MFHAS1 is a regulator of macrophages polarization and CRC development (79).

Macrophages may play an important role in a process called epithelial–mesenchymal transition (EMT). Studies demonstrated that M2-polarized TAMs promoted EMT in pancreatic cancer cells, suggesting a novel mechanism by which M2-polarized TAMs may contribute to the aggressive behavior of pancreatic cancer cells by TLR4/IL-10 signaling (80).

### Modulation of Cancer Microenvironment As Pharmacological Therapy Target

Many factors can contribute to tumorigenesis, tumor progression, and metastasis. Amongst these, the tumor microenvironment is crucial when determining cellular behavior and secretion pattern. Macrophages present in the tumor microenvironment can display different phenotypes as a product of the cytokines they are exposed to. TAMs represent a prevalent hybrid population within tumor microenvironment which usually exhibit an M2-like phenotype (81). The tumor microenvironment and its components, TAMs and their M2-polarized characteristics included, have been extensively studied as therapeutic targets both in conventional treatments and innovative ones.

Morphine is a traditional opioid drug used in clinical practice for pain management of cancer patients. However, it has been discussed whether its effects boost or hinder tumor growth and metastatic behavior (82). Khabbazi et al. developed an approach to enlighten the role morphine treatment might play on the modulation of the tumor microenvironment. Macrophages RAW264.7 and bone marrow derived were polarized either using IL-4 or 4T1 breast cancer cells, as a paracrine communication, toward the M2 profile. Thus, there was a significant escalation in MMP-9 expression, which can uphold invasiveness and angiogenesis. Morphine was shown to prevent said increase, and, therefore, it was hypothesized that the drug might be able to inhibit matrix degradation in the tumor microenvironment. The opioid also lowered arginase-1 expression, which can also be beneficial since its activity boosts breast cancer cell proliferation (83, 84).

Along with new found mechanisms for commonly used drugs, intravenous immunoglobulin (IVIg) is a treatment licensed for a variety of chronic autoimmune diseases such as Kawasaki’s disease and Idiopathic thrombocytopenia purpura, and used “off-label” for a number of other conditions (85). Studies show that IVIg can either have anti-inflammatory action (as expected in clinical practice) on M1 macrophages, or induce phenotype shift from M2 to M1. The drug can elicit either pro- or anti-inflammatory reactions, conditional to the state of macrophage polarization. It has been demonstrated that IVIg significantly hindered secretion of TNF-α, IL-12p40, and CCL2 after M1 macrophages were stimulated with LPS without, nonetheless, affecting their property to inhibit tumor growth. Curiously, when M2 macrophages received the same treatment, the expression of TNF-α, IL-12p40 was boosted. IVIg is now considered to be an immunomodulatory agent, whose effects vary according to the status of the macrophages treated. Such modulatory properties can be extremely valuable in cancer treatments, for it has been demonstrated that IVIg halted tumor progression and metastasis in a model of tumor-associated myeloid cells (86). Another new discovery about an old ally is the evidence that administration of moderate doses of dopamine challenges tumor growth and contributes to vascular stabilization in a rat C6 glioma model. This new property found by Qin et al. can influence TAMs with M2 profile toward the M1 phenotype. Dopamine boosts vascular normalization in tumors by reprogramming M2 macrophages partly *via* dopamine receptor 2 and partly *via* downregulation of the VEGF/VEGFR2 pathway (87).

Moreover, regarding the immunomodulatory properties of substances which can promote polarization of macrophages, the research of Shiri and collaborators can also be highlighted. Their study used curcumin, a fat-soluble substance extracted from *Curcuma longa*, which was bioengineered to enhance its bioavailabitity into DNC-dendrossomal curcumin (88). The DNC formulation has promoted beneficial effects on a murine metastatic breast cancer model, providing an enhanced population of M1 macrophages in detriment of the M2 population (89).

Approved treatments are different in every country, depending on their culture and technological development. For instance, cantharidin is one active ingredient of blister beetles, which is used traditionally in China for cancer treatment (90). Due to toxicity, a demethylated molecule was engineered, the norcantharidin (NCTD), which is routinely used against hepatoma, colorectal carcinoma and breast cancer (91, 92). An interesting new found feature regarding NCTD is its ability to suppress tumor growth through conversion of M2 polarized macrophages to M1 phenotype in a model of murine hepatoma. TAMs extracted from murine tissue of hepatocellular carcinoma could prevent further tumor invasion. When the pathway was investigated, results suggested that the modulation of macrophage polarization by NCTD is somewhat mediated by miR-214 overexpression. TAMs from the same study showed downregulation of STAT3 phosphorylation, a mechanism that has been implicated in the shift of macrophage polarization from M2 to M1 in order to inhibit tumor growth and metastasis (93, 94). In fact, the STAT3 signaling pathway is now considered to be a crucial molecular target when it comes to pancreatic cancer. Arpin et al. have made that evident with their study using a 3D pancreatic ductal adenocarcinoma model and p-STAT3 inhibitors, which was effective even alongside CAFs. They suggest further study on STAT3 inhibition using the same model as an important tool to uncover alternative pathways to be explored in pancreatic cancer treatment (95).

One of the most traditional Chinese medicine, emodin is a natural anthraquinone obtained from *Rheum palmatum L* and other Chinese herbs (96). Interestingly, studies have confirmed that Emodin subdues breast cancer lung metastasis hindering the recruitment and polarization of macrophages toward M2 phenotype in metastatic lung cancer. The substance had no influence over the primary tumor growth, however. Noted, TAMs have a particular subpopulation called metastasis-associated macrophages, which can support metastatic growth (97–99). These cells are also a potential therapeutic target, since metastasis is generally non-responsive to current treatments. Another finding is that the regulation Emodin exhibited over macrophage polarization is through the p-STAT6 and C/EBPβ pathways (100).

The bioflavonoid Wogonin, isolated from *Scutellaria radix*, presents an antitumor properties based on its ability to modulate the tumor microenvironment. In previous studies, Wogonin showed activity through downregulation of MMP-9, which halted invasion of human breast carcinoma cells (101). Wogonin also prevented migration of A549 cells from human alveolar adenocarcinoma, which were used to study EMT, and, therefore, metastasis. The pathway involved in such response was once more *via* IL-6/STAT3, and Wogonin prevented the transition, which demonstrates its potential activity in metastasis (102).

Yet another plant extract shown to have modulation properties on macrophage polarization is isoliquiritigenin (ISL), a flavonoid from licorice. Is has been demonstrated that this substance acts in a chemo preventive manner *via* inhibition of M2 macrophage polarization, in a AOM/DSS-induced colitis-associated tumorigenesis (both *in vivo* and *in vitro*). In this case, the modulation occurs through downregulation of the interaction among IL-6 and PGE_2_. Furthermore, the results also proved a diminished COX-2 expression after stimulation with ISL and a decreased presence of M2 markers such as arginase-1 and CD206 in the lineage macrophages. Evidence showed that ISL inhibited M2 polarization at least partially by the downregulation of the IL-6/STAT3 pathway (103).

Another innovative research regarding modulation of macrophage polarization are cancer vaccines made of *Listeria monocytogenes*. The bacteria are attenuated by deletion of virulence factors, for instance, ActA and Internalin B (ΔactA/ΔinlB) (104). Studies have indicated that treatment with this attenuated strain targets mainly TAMs and reprograms them from M2 phenotype to M1, which activates antitumor responses (105). Results showed that the antitumor function of reprogrammed TAMs was due to nitric oxide production *via* Nos2 (105, 106). This antitumor response mediated by iNOS in repolarized macrophages is so strong that even in the absence of NK cells and CD8 T cells, viability is still maintained (105).

On the other hand, nanotechnology has also been developed to respond to several new-found cancer targets. Carbosilane dendrimer 2G-03NN24 can cause polarization M2 to M1 polarization, decreasing the expression of several M2-related genes, which, in turn, increases the expression of M1 characteristics. These results were confirmed by functional assays, given the M1-oriented proinflammatory and antitumor responses (107). The mechanism involved inhibits phosphorylation of STAT3, which boosts the capacity of macrophages to promote cytotoxic response and presentation of tumor antigens (107, 108). Furthermore, it has been demonstrated that a system composed of gold nanoparticles adapted with MUC-1 protein fragment was capable of promoting macrophage activation and M1 polarization, characterized by increased antigen presentation and M1-like cytokine expression (109). This mucin glycoprotein, MUC-1 or CD227, has been found to be a target for cytotoxic T lymphocytes, and possibly for immunotherapy, hence its use in a possible cancer vaccine (109, 110). Of note, the impact of chemotherapy has been investigated to ascertain what can influence recurrence of cancer. In a study of MCF-7 cells, after apoptosis induced by chemotherapy, there was activation of cancer stem-like cells (CD44+/CD24−) and upregulation of MUC-1 (111). Furthermore, it has been further demonstrated that, when macrophages were cocultured with MCF-7 cells suffering apoptosis, they secreted IL-6, activating STAT3 phosphorylation, and therefore performed a role on the activation of cancer stem cells and cancer promotion. Thus, there is accumulating evidence that blockage of the IL-6/STAT3 pathway is an interesting new approach for metastatic cancer after chemotherapy (112).

Regarding resistance to chemotherapy, there is an increasing body of evidence suggesting the inherent role of the tumor microenvironment in providing cancer cells with means to do so. In this matter, SFRP2 has been identified as Wnt pathway regulator secreted by fibroblasts and an active agonist of WNT16B, which intermediates cancer sensitivity to chemotherapy (113). It has been reported that SFRP2, secreted by stromal cells, triggers the canonical *Wnt* pathway and increases the WNT16B signaling, supporting resistance to chemotherapy. Furthermore, this effect can be abrogated by treatment with antibody as an adjuvant to the chemotherapy. Interestingly, this study considers WNT16B to be a novel biomarker to monitor the response of the tumor microenvironment to therapy (114).

Remarkably, it has increased the number of studies that emphasizes the crucial role of the crosstalk between the adipose tissue and cancer, as an important immunopharmacological target to tumor microenvironment, especially in breast cancer. Qin et al. explored that link by demonstrating the high fat diet cancer latency in C3(1)-Tag GEMMs, which is considered a coherent model to study basal-like breast cancer, was postponed by weight loss when the mice were swapped to low fat diet instead. These interventions could effectively prevent atypical ductal hyperplasia and ductal carcinoma *in situ* to the points observed in healthy mice. In the same study, the weight loss caused decreased activity of several kinases, which, in turn, caused abrogation of the MAPK pathway, which is related to tumor proliferation (115). Another study set out to explore this connection, studying breast cancer cells with overexpression of the HER-2 protein treated with trastuzumab, particularly regarding its ADCC mechanism, that is, the antibody-dependent cellular cytotoxicity. In this setting, it was demonstrated that not only adipocytes and preadipocytes prevented trastuzumab caused NK cell-tumor lysis *in vitro* but also adipose tissue abrogates the drug effect *in vivo* (116). Such effect can be expected once it has been reported that NK cell functions and characteristics can be swayed by the tumor microenvironment (117, 118). Interestingly, they not only display receptors for adipokines but also can have their cytotoxicity modulated by leptin and adipotectin (119, 120). Therefore, the results of Duong et al. suggest that factors expressed by the adipose tissue are responsible for decreasing the sensitivity of HER-2 expressing breast cancer cells to trastuzumab. However, despite testing for several adipokines such as leptin, adiponectin, IL-6, and TNF-α, none were able to account for the ADCC inhibition observed (116).

The investigation of the crosstalk between adipose tissue and breast cancer led to interesting findings, using 3T3-L1 adipocytes and 4T1 breast cancer cells as a model and aspirin as the intervention. It has been demonstrated that aspirin inhibited the proliferation and migration of 4T1 cells and blocked communication between the two cell types *in vitro*, effect observed by the decrease in MCP-1 and PAI-1 expression in the cultured supernatant. Therefore, aspirin is expected to suppress the proliferation of breast cancer cells in an environment shared with obesity (121).

Given the influence the adipose tissue has on cancer promotion, adipokines such as leptin are investigated to uncover their role on the matter. For instance, leptin and its receptor have been demonstrated to facilitate the interaction between cells from the tumor microenvironment, such as cancer-associated fibroblasts and adipocytes, and breast cancer stem cells (BCSCs). Both the pharmacological inhibition of leptin signaling and immunodepletion in the conditioned media used in the experiment both yielded the same result: decreased mammosphere formation in cells obtained from patients with metastatic breast cancer. Therefore, aiming for either leptin or signaling mediated by leptin receptor might eliminate BCSCs or even avert recurrences and metastasis completely in breast cancer patients (122). In a similar study considering the effect of leptin on TAMs and breast cancer cells, the adipokine has been shown to induce IL-18 expression on both, eventually leading the breast cancer cells to invasion and metastasis. In order to confirm the results, cells were co-incubated with IL-18 siRNA or IL-18BP-Fc chimeras and the effect of TAMs to promote migration and proliferation of breast cancer cells when stimulated by leptin was abrogated, thus proving the link between the process (123).

## Conclusion

Adipocytes, macrophages, and tumor cells can orchestrate several signaling pathways that are crucial to the establishment of a microenvironment that can favor cancer progression. Immunomodulatory factors or free-fatty acids released by adipocytes in lipolysis process can fuel tumor cells proliferation, trigger macrophages activation and polarization and control the fate of cancer development. Adipocytes and macrophages regulate themselves, generating an anti-inflammatory tumor microenvironment, preventing immune antitumor response cells from reaching the cancer site. Thus, abundance of white adipocytes and TAM with M2 profile can indicate poor prognosis for patients. Hence, the intimate signaling pathways triggered by those cells can function as a valuable pharmacological target to develop more efficient cancer treatments and should be further explored.

## Author Contributions

LC prepared the main body of the manuscript, figure, and the table. RC, CF, and LD wrote different sections of the manuscript. KM revised, wrote, and prepare the manuscript. All authors listed have made a substantial, direct, and intellectual contribution to the work and approved it for publication.

## Conflict of Interest Statement

The authors declare that the research was conducted in the absence of any commercial or financial relationships that could be construed as a potential conflict of interest.
